# Mitogenomics of the Speartooth Shark challenges ten years of control region sequencing

**DOI:** 10.1186/s12862-014-0232-x

**Published:** 2014-11-19

**Authors:** Pierre Feutry, Peter M Kyne, Richard D Pillans, Xiao Chen, Gavin JP Naylor, Peter M Grewe

**Affiliations:** Charles Darwin University, Research Institute for the Environment and Livelihoods, Ellengowan Drive, Darwin, 0909 Northern Territory Australia; CSIRO Oceans and Atmosphere Flagship, 41 Boggo Road, Dutton Park, 4102 Queensland Australia; Guangxi Key Lab for Mangrove Conservation and Utilization, Guangxi Mangrove Research Center, Guangxi Academy of Sciences, Beihai, 536000 PR China; Department of Biology, College of Charleston, Charleston, SC 29412 USA; CSIRO Oceans and Atmosphere Flagship, Castray Esplanade, Hobart, 7000 Tasmania Australia

**Keywords:** Population genetics, Elasmobranchs, Philopatry, IUCN Red List, Dispersal, D-loop, Low genetic diversity

## Abstract

**Background:**

Mitochondrial DNA markers have long been used to identify population boundaries and are now a standard tool in conservation biology. In elasmobranchs, evolutionary rates of mitochondrial genes are low and variation between distinct populations can be hard to detect with commonly used control region sequencing or other single gene approaches. In this study we sequenced the whole mitogenome of 93 Critically Endangered Speartooth Shark *Glyphis glyphis* from the last three river drainages they inhabit in northern Australia.

**Results:**

Genetic diversity was extremely low (π =0.00019) but sufficient to demonstrate the existence of barriers to gene flow among river drainages (AMOVA *Φ*_ST_ =0.28283, *P* <0.00001). Surprisingly, the comparison with single gene sub-datasets revealed that ND5 and 12S were the only ones carrying enough information to detect similar levels of genetic structure. The control region exhibited only one mutation, which was not sufficient to detect any structure among river drainages.

**Conclusions:**

This study strongly supports the use of single river drainages as discrete management units for the conservation of *G. glyphis*. Furthermore when genetic diversity is low, as is often the case in elasmobranchs, our results demonstrate a clear advantage of using the whole mitogenome to inform population structure compared to single gene approaches. More specifically, this study questions the extensive use of the control region as the preferential marker for elasmobranch population genetic studies and whole mitogenome sequencing will probably uncover a large amount of cryptic population structure in future studies.

**Electronic supplementary material:**

The online version of this article (doi:10.1186/s12862-014-0232-x) contains supplementary material, which is available to authorized users.

## Background

Genetic markers have long been used to discriminate between populations and define evolutionary significant units [1] or management units [2], including for marine or euryhaline species for which barriers to migration are rarely easy to identify [3,4]. The choice of marker employed is critical as it has a strong influence on the ability to identify discrete populations. Markers with low mutation rates could prevent or reduce the probability of detecting population differentiation, even in truly non-panmictic populations [5]. Nearly three decades ago, Avise & Ellis [6] advocated using mitochondrial DNA (mtDNA) genes as preferential markers in phylogeographic genetic studies. Mitochondrial genes have since proved to be useful markers to infer species or population boundaries, and have become a standard tool in conservation biology [7,8].

Progeny generally inherit their mtDNA from the female parent. As such, the analysis of mtDNA reflects matriarchal lineage processes only, providing information unobtainable from any nuclear marker [9]. This is particularly important in groups such as elasmobranchs (sharks and rays), where there is accumulating evidence of reproductive philopatry in females but not in males [10-12].

Mitochondrial DNA markers have been widely used in studies of teleost and elasmobranch fish populations over the past decade with much attention focused on the D-loop or control region (CR). This region has been shown to be extremely variable in many vertebrates including humans [13], teleosts [14] and birds [15]. In elasmobranchs however, evolutionary rates of mitochondrial genes are low compared to other vertebrates [16], and variation between distinct populations can sometimes be hard to detect with commonly used CR sequencing approaches [17,18]. However, a thorough examination of intraspecific rates of evolution of the different mtDNA regions in sharks and rays has not yet been carried out, and there may be alternative regions exhibiting useful polymorphisms that could be exploited for population genetic analysis. Interestingly, the highest single gene mitochondrial nucleotide diversity reported in any elasmobranch species so far was in the Dark Shyshark *Haploblepharus pictus* using the COXI gene [19] followed by the Discus Ray *Paratrygon aiereba* using the ND6 gene [20]. Also, Naylor et al. [21] collected 4,283 ND2 sequences and looked at intraspecific divergences in 595 different elasmobranch species. Many were found to show substantial differences among populations. In order to increase the ability to detect population structure, a few studies have included the analysis of more than one mtDNA marker with variable success [17,22,23]. One study included a preliminary screening of four different regions to find the most variable one, which happened to be the ND4 gene followed by the ATP gene and then the CR [24]. These results suggest that in elasmobranchs the CR could be under higher evolutionary constraints (compared to other mitochondrial regions) than in other vertebrates.

Recent advances in next generation sequencing have significantly reduced costs in a way that could easily facilitate routine investigation of intraspecific variation of whole elasmobranch mtDNA genomes at typical sampling levels used for standard population analyses. In the past, whole mitogenome sequencing has only been used in phylogenetic studies, where the number of individuals sequenced was generally low. Compared to single gene approaches, whole mitogenome sequencing can sometimes provide higher resolution of phylogenies and increased precision of divergence time estimates [25]. More recently, whole mitogenome sequencing has been used to investigate diversity at the intra-species level. For example, it provided improved resolution of population structure in the Green Turtle *Chelonia mydas* [26] and the North American Fisher *Martes pennanti* (a weasel) [27]. It has also been used in species delineation [28] and the detection of genes under selection in the Killer Whale *Orcinus orca* [29]. There are currently no publications using this method in elasmobranchs.

The Speartooth Shark *Glyphis glyphis* is a rare, habitat- and range-restricted, river shark of northern Australia and southern Papua New Guinea [30]. It is confirmed from only eight rivers of northern Australia, with the majority of records coming from Van Diemen Gulf drainages (Adelaide, South Alligator, and East Alligator Rivers) in the Northern Territory, and the Wenlock River, Western Cape York Peninsula, Queensland. There is an apparent large gap (>1,000 km) in its distribution between the Van Diemen Gulf drainages and the Wenlock River [30]. Its rarity, limited range, and the suspected impacts of fishing activities resulted in a listing of Critically Endangered on the Australian *Environment Protection and Biodiversity Conservation Act* 1999. Little is known of its biology: juveniles and sub-adults have been recorded across a large salinity range in rivers, however, adults of this species have never been recorded and their range and habitat requirements are unknown [30]. An understanding of population structure is required to inform management and conservation measures for this species. Wynen et al. [17] developed a DNA barcoding approach for confirming species delineation using portions of the COX1 gene and the CR. This approach proved to be successful in distinguishing between *G. glyphis* and the sympatric Northern River Shark *G. garricki* and Bull Shark *Carcharhinus leucas*, but no intraspecific variation was found across 12 and 17 *G. glyphis* individuals from the Northern Territory and Queensland, respectively, thus preventing any assessment of potential population structure.

The aim of this study was to explore the potential of whole mitogenome sequencing for phylogeographic studies in elasmobranchs, using *G. glyphis* as a case study. We anticipated that this approach would provide useful information for the conservation of this Critically Endangered species, as well as giving insights into the intraspecific genetic variability of the different mitogenomic regions in elasmobranchs.

## Results

### Data reliability

The complete mitogenomes of 93 *G. glyphis* were successfully sequenced and assembled (Genbank Accessions, KM100613 - KM100704). Individual mean coverage ranged from 152 to over 6,000 fold ensuring a high quality of mitogenome sequencing. The duplicated sequencing of the *G. glyphis* reference mitogenome [31] provided the exact same sequence twice, demonstrating a high degree of reliability in the dataset.

Less than 0.5% of the reads from *G. glyphis* samples were successfully mapped on the Largetooth Sawfish *Pristis pristis* mitogenome, the other elasmobranch species present in the Miseq run. Those reads were mostly mapped on the highly conserved 16S region, thus suggesting cross contamination did not occur during library preparation.

### Mitogenomic diversity

**Figure 1:**
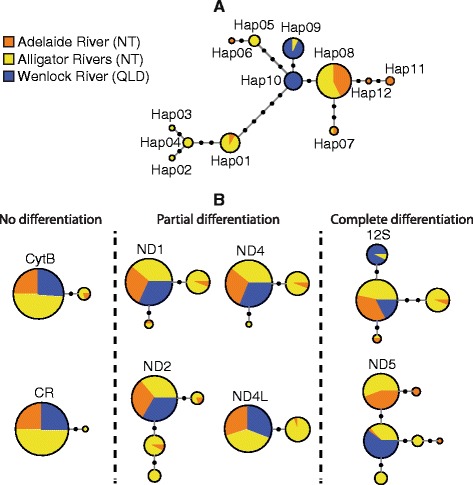
**Haplotype networks inferred from (A) whole mitogenome dataset; (B) single region sub-datasets.** Black circles on connecting lines between haplotypes represent single mutation steps for haplotypes not found in our current data.

Twelve haplotypes were observed among the 93 fish from the three river drainages sampled (Figure 1). No insertions or deletions were observed, and the *G. glyphis* mitogenome was calculated to be 16,701 bp in length. Interestingly, no transversions were observed but there were 19 transitions spread across 12 haplotypes. Nucleotide diversity (π) was 0.00019 and haplotype diversity (H_d_) 0.76. Only one amino acid change was found in the complete dataset, at position 1348 of the ND5 alignment; this was not river drainage specific. The highest genetic diversity was found in the Alligator Rivers (π = 0.00024 and H_d_ = 8), whereas the Wenlock River exhibited the lowest genetic diversity (π = 0.00003 and H_d_ = 2). River drainages each contained from one to four drainage-specific haplotypes (Figure 1). Genetic diversity indices for each of the drainage locations derived from the whole genome dataset are given in Table 1.

**Table 1:** **Summary of genetic diversity indices inferred from whole mitogenomes across the study sites and for each of the three river drainages sampled**

**Diversity indices**	**All samples**	**Adelaide River**	**Alligator Rivers**	**Wenlock River**
π	0.00019	0.00009	0.00024	0.00003
Variable sites - S	19	14	16	1
Singleton sites	3	11	5	0
Haplotypes	12	6	8	2
H_d_	0.760	0.458	0.685	0.514
Tajima’s D	-0.42548	-2.11498	0.32904	1.53267
Significance	P > 0.10	P=0.001	P > 0.10	P > 0.10
Fu’s F_s_	-0.22900	-0.79791	2.18415	1.52985
Significance	P > 0.10	P > 0.10	P > 0.10	P > 0.10

The ‘all proteins’ sub-dataset recovered 13 out of the 19 variable sites. Among the single gene sub-datasets, ND5 was the most diverse with 5 variable sites and a nucleotide diversity of 0.00042, followed by ND2 with 3 variable sites and a nucleotide diversity of 0.00051. The 12S region harbored 4 variable sites for a nucleotide diversity of 0.00101. Several sub-datasets, including 16S, COX1, COX2, COX3, ND3, ND6, ATP6 and ATP8 did not exhibit any variation. Additional file 1: Table S1 summarizes the variable site positions per mitogenome region and per haplotype.

With the exception of Tajima’s D in the Adelaide River, neutrality tests calculated from the whole mitogenome were not significant (Table 1). Neutrality tests calculated from single region sub-dataset were generally concordant with this result. A complete set of genetic diversity indices for the whole mitogenome and all sub-datasets is available in Additional file 2: Table S2.

### Population structure

Significant genetic structure was detected among the three river drainages (AMOVA *Φ*_ST_ = 0.28283, *P* <0.00001). The pairwise differentiation *Φ*_ST_ values ranged from 0.17755 to 0.53088 and all were significant (Table 2). The genetic differentiation inferred from each sub-dataset varied greatly (Additional file 3: Table S3). Apart from the ‘all proteins’ sub-dataset, only two single region sub-datasets, 12S and ND5, recovered significant Φ_ST_ values for all population pairs. ND1, ND2, ND4 and ND4L suggested the existence of barriers to gene flow between the Adelaide River and the Alligator Rivers, and between the Alligator Rivers and the Wenlock River, but not between the Adelaide River and the Wenlock River. No signs of genetic differentiation were found in CytB and CR, the remaining sub-datasets that exhibited some degree of genetic variation. Interestingly, overall values of Φ_st_ appeared concordant with geographical distances among the three populations, with Adelaide-Alligators (Φ_st_ = 0.18) having lower values than the Alligators-Wenlock and Adelaide-Wenlock (Φ_st_ = 0.30 and 0.53, respectively).

**Table 2:** **Pairwise Φ**_**st**_ **(below) and associated p-values (above) for population comparisons derived from the whole mitogenome dataset**

**Population**	**Adelaide River**	**Alligator Rivers**	**Wenlock River**
**Adelaide River**	-	0.00168	< 0.00001
**Alligator Rivers**	0.17756	-	< 0.00001
**Wenlock River**	0.53088	0.30185	-

## Discussion

The whole mitogenomic survey using next generation sequencing (MiSeq, Illumina) proved to be an efficient and accurate method for routine surveys of *G. glyphis* population structure in Australia. All mitogenomic sequences were easily amplified by PCR and sequenced with a high degree of reliability in a time-efficient manner. The data collection pipeline, from PCR amplification to the mitogenome alignment ready for analysis, could be completed in one week if all individuals are sequenced on the same Miseq run. Despite some discrepancies in coverage across samples, the high read coverage provided by the Miseq ensured sufficient coverage for each individual. Additionally, the attempt to map reads from *G. glyphis* barcodes on the *P. pristis* reference mitogenome demonstrated that cross contamination of sequence data was not significant. Following the method described here, whole mitogenome sequencing is currently 4-5 fold more expensive than single fragment analysis by Sanger sequencing. However, this method could be optimized in several ways to make the best use of the high number of reads offered by high throughput sequencers, including (i) pooling more libraries on a given sequencing run [32]; or (ii) pooling several species in each library [33]. Whole mitogenome sequencing costs will also continue to fall as technology improves.

Mitogenomic sequencing in *G. glyphis* revealed particularly low levels of genetic diversity. Previous to this study, the species that exhibited the lowest recorded genetic diversity inferred from a whole mitogenome was the Giant Squid, *Architeuthis* sp., in a survey of 43 individuals from 10 sampling locations [34]. This species harbored a nucleotide diversity of 0.00066, more than three times higher than that of *G. glyphis*. The low diversity in the Giant Squid is believed to be due to a recent bottleneck followed by expansion, possibly coupled with a low mutation rate [34]. With the exception of the Adelaide River, there were no signs of recent population expansion in *G. glyphis*, suggesting that a bottleneck followed by population expansion is unlikely to be the best explanation for this species.

A low mutation rate, however, could partly explain such low diversity: rates of mitochondrial DNA evolution in elasmobranchs are slow compared to other taxa [16]. To the best of our knowledge, there is no other study investigating the intraspecific variability of whole mitogenomic sequences in elasmobranchs to compare with our data. However, our review of the current literature examining single gene analysis of mitochondrial diversity in sharks and rays (Additional file 4: Table S4) indicates that slow mutation rate alone cannot completely explain the low nucleotide diversity found in *G. glyphis*. All species included in this review had populations with higher nucleotide diversity indices; 42 out of 50 species exhibited nucleotide diversity indices at least one order of magnitude higher than *G. glyphis* (Additional file 4: Table S4). Some shark species however, exhibited populations without any or only very low levels of nucleotide diversity for the gene under study. Again, a recent bottleneck event was proposed to explain the low genetic diversity observed in the Basking Shark *Cetorhinus maximus* [35] and the Nurse Shark *Ginglymostoma cirratum* [36], two coastal species with large ranges. In the Grey Nurse Shark *Carcharias taurus*, demographic events in the deep history of the species (bottlenecks or founder effects) and especially low levels of molecular evolution were proposed to explain current levels of genetic diversity [37]. In addition to past demographic events, current effective population size can also affect levels of nucleotide diversity [38,39]. *Glyphis glyphis* has a naturally very limited range, being recorded in only a small number of tidal rivers and their estuaries in northern Australia and southern Papua New Guinea resulting in a low expected total number of individuals for this species [40]. We thus argue that the low genetic diversity observed in *G. glyphis* is probably due to the low evolutionary rate of mitochondrial genes (common in elasmobranch species, [16]) combined with an extremely small population size leading to subsequent loss of diversity through genetic drift [41]. These factors also apply in *G. garricki*, which shares with *G. glyphis* its limited habitat, limited range and probably small localized populations. This could explain the absence of genetic variation in the CR of 15 *G. garricki* analysed by Wynen et al. [17]. Low levels of genetic diversity were also observed in *C. leucas* in northern Australia, another species sharing juvenile life cycle and habitat characteristics with *G. glyphis* [23].

Despite very low observed genetic diversity among river drainages, our whole mitogenome sequencing approach was able to detect sufficient variation to provide the first insight into the population structure of *G. glyphis*. Haplotype diversity appears to be partitioned in a way that suggests strong female philopatry as each of the three river drainages is home to a distinct genetic population. Prior to our study, no evidence of population structure had been observed, however this was inferred from single gene approaches [17]. Our result is critical for the implementation of conservation measures to manage and ultimately recover this Critically Endangered species. Tissue samples of *G. glyphis* used in this study were obtained from juvenile and sub-adult animals captured in the Adelaide, Alligators and Wenlock River drainages. In Australia, this represents the entire known extant distribution of *G. glyphis* with the population in the Bizant River, Eastern Cape York Peninsula presumed extinct [30]. Given the population structuring and low levels of female dispersal highlighted in this study, remaining population centers need to be managed as discrete population units. The life history of *G. glyphis* is not fully understood, although given the apparent absence of adults from river systems, adults presumably occur in marine habitats [30]. The presence of a boundary to gene flow in females indicates that females return to the river of their birth to undertake parturition, but whether adult populations are also segregated remains unknown. As mtDNA strictly reflects female-mediated gene flow, further investigation of nuclear markers is warranted to evaluate patterns of male-mediated gene flow among these populations. In *C. leucas,* population structuring occurred only at very large scales and not between adjacent rivers [23]. *Glyphis glyphis* either has more discrete reproductive habitat preferences than *C. leucas* (at least for females) or the two gene approach used by Tillett et al. [23] did not have the power to detect structure at a finer scale, as would have been the case in *G. glyphis* using a similar approach (Figure 1; Additional file 3: Table S3).

The analysis of each sub-section of the mitogenome further highlighted the advantage of whole mitogenome sequencing compared to single gene approaches. Several commonly used mitogenome regions, including CR and COX1 were invariant and hence failed to detect any barrier to gene flow between the three river drainages under study. These results were consistent with previous work on *G. glyphis* by Wynen et al. [17]. In the current study, ND1, ND2, ND4 and ND4L genes all provided partial insights into the population structuring of *G. glyphis*. A similar finding was made in *Chelonia mydas*, where whole mitogenomic sequences improved the population structure resolution compared to single gene analysis [26]. Interestingly, the strongest barrier to gene flow inferred from the whole mitogenome dataset (between the Adelaide River and the Wenlock River), was the most difficult to identify with single region datasets. This is because the haplotypes harbored by sharks from the Wenlock River differ from the most common haplotype by 1 or 2 mutations only. The uniqueness of those haplotypes relies on a very low number of mutations (Figure 1), clearly demonstrating the difference that can be made by sequencing the whole mitogenome compared to a subset of the mitogenome only. Not sequencing the mitogenome sections containing those critical mutations would have missed this population structure pattern. This has particularly important ramifications for species with low mutation rates such as elasmobranchs or in cases where isolation between populations has only occurred recently [26].

In *G. glyphis*, 12S and ND5 were the most variable mitochondrial regions and the only ones providing comparable resolution to that of the whole mitogenome to detect barriers to gene flow between each river drainage pair. This result challenges the assumptions commonly made over the past decade on which marker should be used in elasmobranch phylogeographic studies. Our literature review revealed that the CR was the most commonly chosen marker with ND4 and CytB the two next most common (CR, 40 studies; ND4, 7 studies; CytB, 6 studies) (Additional file 4: Table S4). In the absence of any comprehensive study investigating intraspecific rates of evolution in the different mitogenomic regions in elasmobranchs, we argue that the wide use of the CR in elasmobranchs relies mostly on an assumption that this region will be the most variable in the mitogenome, as observed in other taxa [13]. In fact, the few times CR has been compared to other regions, it was not found to be most variable [22,24]. We do not argue that the CR is always a poor marker for phylogeographic studies in elasmobranch species and should not be used. For example, at least five studies analyzing the CR in elasmobranch species report nucleotide diversity indices over 0.01 thus providing a good capacity to detect population structure [42-46]. Also, many other studies were successful in detecting some degree of population structure despite lower levels of nucleotide diversity in the CR (Additional file 4: Table S4). However, our study demonstrates clearly that other regions of the mitogenome should be examined as they potentially improve population resolution.

The 12S and ND5 regions are the two most variable markers reported here, but they have not previously been used for intra-specific phylogeographic studies in elasmobranchs. The 12S region, however, is often used to infer phylogenetic relationships between elasmobranch species [47] or for species identification [48]. Similarly, the ND5 gene has been rarely used in phylogeographic studies of elasmobranchs, although see [49] for *Manta* species delineation. Analysis of other elasmobranch species is required to further investigate the potential of 12S and ND5 as a source of mitochondrial intraspecific diversity.

## Conclusions

The current study clearly demonstrates the difference that whole mitogenome sequencing can make in population genetic studies that focus on species with low genetic diversity. Given the current state of technology and sequencing costs, we strongly recommend the use of whole mitogenome sequencing for future population genetic studies in elasmobranchs. Intraspecific mitogenomic surveys may expose a large amount of cryptic population structure in species that have only been examined for a single mitochondrial region such as the CR. At a minimum, we suggest preliminary whole mitogenome analyses on a small number of individuals should be performed to identify the more variable mitochondrial regions when population-level sequencing is cost-prohibitive. Forthcoming mitogenomic surveys might also help to identify more general patterns of variability permitting a better choice of specific mitochondrial genes containing variable regions of interest in elasmobranchs.

## Methods

### Sampling, DNA extraction and long-range PCR amplification

**Figure 2:**
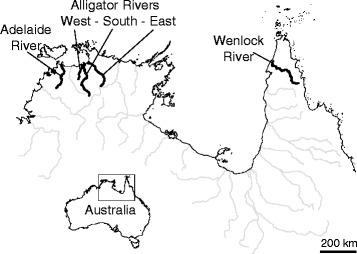
**River drainages (highlighted in bold lines) indicating approximate sample collection origins in northern Australia.**

Ninety-three *G. glyphis* were sampled from the Adelaide River (n = 23), the combined (South, East and West) Alligator Rivers (n = 47) (referred to as the Alligator Rivers) in the Northern Territory, and the Wenlock River (n = 23) in Queensland, Australia (Figure 2). This represents the entire extant northern Australian distribution of the species, with the Adelaide River the westernmost occurrence and the Wenlock River the easternmost occurrence [30]. Most sharks were sampled in 2012 and 2013, except six from the Adelaide River, five from the Alligator Rivers and thirteen from the Wenlock River that were sampled as part as a previous study (Wynen et al. [17]). Sharks ranged from neonates with open umbilical scars to sub-adults up to 156 cm TL, indicating that our samples comprised multiple year classes.

DNA was extracted from fin clips using DNeasy Blood & Tissue kits (Qiagen, Venlo, Netherlands). Whole mitogenomes were amplified in two overlapping fragments using Takara LA Taq, a proofreading Taq polymerase mixture (Takara, Otsu, Shiga, Japan). The PCR reaction mix consisted of 1 unit of Takara LA Taq, 2 μl of 10x LA PCR Buffer II (Mg2+ free), 2 μl of 25 mM MgCl2, 3.2 μl dNTP (2.5 mM each), 0.5 μl of each primer at 10 mM, and 10.6 μl sterilized distilled water. The primer pairs GL965F – 5′ TATTTCTCCAACAAGAGGAGGCAAGTCGTAAC 3′/GL10307R - 5′ GTAGTTGGTCAGAACCGTGT 3′ and GL10014F - 5′ GTCCAAATCAAGACCGCTAA 3′/GL1403R – 5′ CTTTTGCCACAGAGACGG 3′ were designed using previously acquired conserved areas of whole mitogenomic sequences of *G. glyphis* [31] and *G. garricki* [50]. PCR conditions consisted of an initial denaturation at 94°C for 3 min followed by 35 cycles of 94°C for 35 sec and 68°C for 10 min, with a final extension step of 72°C for 10 min. PCR amplicons for each individual were purified with Agencourt AMPure XP magnetic beads (Beckman Coulter Inc., Indianapolis, Indiana, USA), quantified with NanoDrop 8000 Spectrophotometer (Thermo Fisher Scientific, Waltham, Massachusetts, USA) and pooled at equimolar concentrations for each individual previous to the preparation of libraries.

### Nextera library preparation and Miseq sequencing

The two purified fragments obtained for each individual were pooled at equimolar concentrations and subjected to library preparation using Nextera XT DNA Sample Preparation kits (Illumina, San Diego, California, USA). Individual Nextera XT libraries were simultaneously fragmented and barcoded using the 96 sample Nextera Index kit (Illumina, San Diego, California, USA). All libraries were quantified by a fluorometric method, using the Qubit dsDNA HS assay kit (Life Technologies, Carlsbad, California, USA), and concentrations were normalized to give equal mitogenome coverage from each individual fish. Libraries were then pooled and sequenced on a Miseq desktop sequencer using 2 × 250 bp paired-end reads Miseq reagent kit v2 (Illumina, San Diego, California, USA). The sequencing was done in two different runs, approximately half of the samples for each run being from *P. pristis*, another elasmobranch species (data not shown).

### Mitogenome assembly and data analysis

Fastq files were imported into GENEIOUS PRO (v. 7.0.6) (Biomatters Ltd., Auckland, New Zealand). Before mitogenome assembly, reads were paired; 5′ and 3′ ends as well as regions with more than 5% chance of an error per base were trimmed. Reads with more than 15 low quality bases and/or shorter than 150 bp after trimming were discarded from subsequent analyses. Reads for each individual were then mapped onto a previously published reference sequence [31] using ‘Map to Reference’ tool in GENEIOUS PRO without fine-tuning and default parameters. PCR duplicates were removed using the rmdup SAMTools toolkit (v. 1.0.0) [51]. The Majority rule consensus (>50% of reads for any single SNP, insertion, deletion) for each fish was exported. Consensus sequences were then aligned using MUSCLE alignment tool with default parameters in GENEIOUS PRO. The mitogenome of the exact same fish sequenced by Chen et al. [31] was amplified, sequenced once in each of the Miseq runs and assembled twice. Replicates were compared to the reference sequence to check for data reliability. As a second quality check, reads from each *G. glyphis* sample were mapped on the reference mitogenome of the second elasmobranch species included in the Miseq sequencing runs.

Preliminary reconstruction of a maximum likelihood haplotype tree indicated that the level of nucleotide variation among haplotypes was too low to provide a well-supported phylogenetic tree (Additional file 5: Figure S1). As an alternative to represent haplotype relationships, we drew a median-joining network [52] with NETWORK (v 4.6.1.2) (Fluxus Technology Ltd, Clare, Suffolk, England) using default parameters (Figure 1A). The same method was employed to draw haplotype networks for each single region sub-dataset (Figure 1B).

A range of genetic diversity indices was calculated for all sites and also each river separately using ARLEQUIN (v 3.5.1.2) [53]. This includes the nucleotide diversity index π [54], the number of variable sites S [55], the number of singleton sites, the number of haplotypes, and the haplotype diversity index H_d_ [54]. Tajima’s D [56] and Fu’s F_s_ [57] statistics were also calculated to infer the demographic history of *G. glyphis*.

Analyses of molecular variance (AMOVA) were conducted with ARLEQUIN (v 3.5.1.2), to test for the existence of genetic structure among animals from different river drainages, and we calculated the fixation index (Φ_ST_) to estimate the degree of genetic differentiation among the three sampling locations. This was done for the whole mitogenome dataset and for each sub-dataset containing variable sites. The model of nucleotide evolution used for the AMOVA and to calculate Φ_ST_ values was Tamura-Nei [58].

A literature review was conducted to evaluate preferences in mitochondrial markers and to estimate their degree of genetic diversity (π) in elasmobranchs (Additional file 4: Table S4). Scopus and Google Scholar were used as the search engines with the following keywords: phylogeography, elasmobranch(s), mitochondrial DNA, shark(s), ray(s), population structure, genetic diversity, genetic(s). This review was not intended to be comprehensive, but to provide a good representation of previous research. For this reason we avoided using marker name (e.g. COXI, 16S or control region) as keywords in order not to bias the review towards a particular marker.

## Availability of supporting data

Mitogenomic sequences: Genbank accessions KM100613 - KM100704.

## Additional files

Additional file 1: Table S1. Variable site positions in *G. glyphis* mitogenome alignment. Additional file 2: Table S2. Genetic diversity indices per river and per mitogenomic region. Additional file 3: Table S3. Pairwise Φ_st_ values derived from each mitogenomic region exhibiting some degree of genetic variation. Additional file 4: Table S4. Mitochondrial nucleotide diversity π in elasmobranchs, literature review summary. Additional file 5: Figure S1. Maximum likelihood haplotype tree.
