# Supplementary material for: Mitogenomics of the Speartooth Shark challenges ten years of control region sequencing
Source: BMC Evol Biol. 2014 Nov 19;14:232. doi: 10.1186/s12862-014-0232-x (PMC4245800; doi:10.1186/s12862-014-0232-x)
Supplement: Additional file 4: Table S4. — Mitochondrial nucleotide diversity π in elasmobranchs, literature review summary. [file 12862_2014_232_MOESM4_ESM.docx]

Table S4. Mitochondrial nucleotide diversity π in elasmobranchs. Regional scale corresponds to indices calculated for populations including samples from a geographic area that extend from a river system up to an ocean basin. Worldwide scale corresponds to indices calculated for populations including samples from at least two ocean basins.

| **Order** | **Family** | **Common Name** | **Species** | **Marker** | **Regional scale** | **Global scale** | **Source** |
| --- | --- | --- | --- | --- | --- | --- | --- |
| Squaliformes | Squalidae | Spiny Dogfish | *Squalus acanthias* | ND2 | 0.0015-0.0054 | 0.0086 | [Veríssimo *et al.* 2010](#_ENREF_42) |
| Squaliformes | Centrophoridae | Leafscale Gulper Shark | *Centrophorus squamosus* | ND2 | 0.0011-0.0020 | 0.0018 | [Veríssimo *et al.* 2012](#_ENREF_43) |
| Squaliformes | Somniosidae | Portuguese Dogfish | *Centroscymnus coelolepis* | CR | 0.0018 |  | [Veríssimo *et al.* 2011](#_ENREF_44) |
| Squaliformes | Somniosidae | Southern Sleeper Shark | *Somniosus antarcticus* | CytB | 0.0023 |  | [Murray *et al.* 2008](#_ENREF_27) |
| Squaliformes | Somniosidae | Greenland Shark | *Somniosus microcephalus* | CytB | 0.0022 |  | [Murray *et al.* 2008](#_ENREF_27) |
| Squaliformes | Somniosidae | Pacific Sleeper Shark | *Somniosus pacificus* | CytB | 0.0031-0.0043 |  | [Murray *et al.* 2008](#_ENREF_27) |
| Rajiformes | Pristidae | Dwarf Sawfish | *Pristis clavata* | CR | 0.0040 |  | [Phillips *et al.* 2011](#_ENREF_31) |
| Rajiformes | Pristidae | Largetooth Sawfish | *Pristis pristis* | CR | 0.0044 |  | [Phillips *et al.* 2011](#_ENREF_31) |
| Rajiformes | Pristidae | Green Sawfish | *Pristis zijsron* | CR | 0.0036 |  | [Phillips *et al.* 2011](#_ENREF_31) |
| Rajiformes | Rhinobatidae | Shovelnose Guitarfish | *Rhinobatos productus* | CR | 0.0106-0.0131 |  | [Sandoval-Castillo *et al.* 2004](#_ENREF_35) |
| Rajiformes | Rhinobatidae | Banded Guitarfish | *Zapteryx exasperata* | CR/ND2 | 0.0011 |  | [Castillo-Páez *et al.* 2014](#_ENREF_5) |
| Rajiformes | Rajidae | Thorny Skate | *Amblyraja radiata* | CytB | 0.009 |  | [Chevolot *et al.* 2007](#_ENREF_8) |
| Rajiformes | Rajidae | Common Skate | *Dipturus batis* | CR | 0.01774 |  | [Griffiths *et al.* 2010](#_ENREF_14) |
| Rajiformes | Rajidae | Longnose Skate | *Dipturus oxyrinchus* | CR | 0.00223 |  | [Griffiths *et al.* 2011](#_ENREF_15) |
| Rajiformes | Rajidae | Thornback Skate | *Raja clavata* | CR | 0.0014-0.0039 |  | [Pasolini *et al.* 2011](#_ENREF_29) |
| Rajiformes | Rajidae | Biscuit Skate | *Raja straeleni* | CR | 0.0025 |  | [Pasolini *et al.* 2011](#_ENREF_29) |
| Rajiformes | Dasyatidae | Red Stingray | *Dasyatis akajei* | CR | 0.0021-0.0084 |  | [Li *et al.*](#_ENREF_23) (in press) |
| Rajiformes | Dasyatidae | Shorttail Stingray | *Dasyatis brevicaudata* | CR | 0.0003-0.0019 | 0.0009 | [Le Port & Lavery 2012](#_ENREF_21) |
| Rajiformes | Potamotrygonidae | Discus Ray | *Paratrygon aiereba* | ND6 | 0.0053-0.0349 |  | [Frederico *et al.* 2012](#_ENREF_11) |
| Rajiformes | Myliobatidae | Spotted Eagle Ray | Aetobatus ocellatus  (as A. narinari) | CytB | 0.0126 |  | [Schluessel *et al.* 2010](#_ENREF_36) |
| Rajiformes | Myliobatidae | Spotted Eagle Ray | Aetobatus ocellatus  (as A. narinari) | ND4 | 0.0085 |  | [Schluessel *et al.* 2010](#_ENREF_36) |
| Rajiformes | Rhinopteridae | Pacific Cownose Ray | Rhinoptera steindachneri | ND2 | 0.00255 |  | [Sandoval-Castillo & Rocha-Olivares 2011](#_ENREF_34) |
| Orectolobiformes | Ginglymostomatidae | Nurse Shark | *Ginglymostoma cirratum* | CR | 0.0001-0.0005 | 0.0008 | [Karl *et al.* 2012](#_ENREF_18) |
| Orectolobiformes | Stegostomatidae | Zebra Shark | *Stegostoma fasciatum* | ND4 | 0-0.0038 | 0.0014 | [Dudgeon *et al.* 2009](#_ENREF_9) |
| Orectolobiformes | Rhincodontidae | Whale Shark | *Rhincodon typus* | CR | 0.004-0.007 | 0.011 | [Castro *et al.* 2007](#_ENREF_6) |
|  |  |  |  | CR | 0.005 |  | [Ramírez-Macías *et al.* 2007](#_ENREF_33) |
| Lamniformes | Odontaspididae | Grey Nurse Shark | *Carcharias taurus* | CR | 0-0.00213 | 0.004 | [Ahonen *et al.* 2009](#_ENREF_1) |
|  |  |  |  | CR | 0-0.003 |  | [Stow *et al.* 2006](#_ENREF_38) |
| Lamniformes | Alopiidae | Pelagic Thresher Shark | *Alopias pelagicus* | CR | 0.00113-0.00360 |  | [Trejo 2005](#_ENREF_41) |
| Lamniformes | Alopiidae | Bigeye Thresher Shark | *Alopias superciliosus* | CR | 0-0.00334 | 0.00166 | [Trejo 2005](#_ENREF_41) |
| Lamniformes | Alopiidae | Common Thresher Shark | *Alopias vulpinus* | CR | 0-0.00149 | 0.00094 | [Trejo 2005](#_ENREF_41) |
| Lamniformes | Cetorhinidae | Basking Shark | *Cetorhinus maximus* | CR | 0.0013-0.0014 | 0.0013 | [Hoelzel *et al.* 2006](#_ENREF_16) |
| Lamniformes | Lamnidae | White Shark | *Carcharodon carcharias* | CR | 0.00855 |  | [Blower *et al.* 2012](#_ENREF_4) |
| Carcharhiniformes | Scyliorhinidae | Dark Shyshark | *Haploblepharus pictus* | COXI | 0.003-0.053 |  | [McLachlan 2011](#_ENREF_24) |
| Carcharhiniformes | Triakidae | School Shark | *Galeorhinus galeus* | CR | 0.001-0.002 | 0.007 | [Chabot & Allen 2009](#_ENREF_7) |
| Carcharhiniformes | Triakidae | Narrownose Smoothhound | *Mustelus schmitti* | CytB | 0.0015 |  | [Pereyra *et al.* 2010](#_ENREF_30) |
| Carcharhiniformes | Triakidae | Leopard Shark | *Triakis semifasciata* | CR | 0.0067 |  | [Lewallen *et al.* 2007](#_ENREF_22) |
| Carcharhiniformes | Carcharhinidae | Pigeye Shark | *Carcharhinus amboinensis* | ND4 | 0.0032-0.0089 |  | [Tillett *et al.* 2012a](#_ENREF_39) |
| Carcharhiniformes | Carcharhinidae | Bronze Whaler | *Carcharhinus brachyurus* | CR | 0.00037-0.00203 | 0.016 | [Benavides *et al.* 2011a](#_ENREF_2) |
| Carcharhiniformes | Carcharhinidae | Spinner Shark | *Carcharhinus brevipinna* | ND4 | 0.0010-0.0016 | 0.0013 | [Geraghty *et al.* 2013](#_ENREF_13) |
| Carcharhiniformes | Carcharhinidae | Silky Shark | *Carcharhinus falciformis* | CR | 0.0009 |  | [Galván-Tirado *et al.* 2013](#_ENREF_12) |
| Carcharhiniformes | Carcharhinidae | Bull Shark | *Carcharhinus leucas* | CR | 0.0028 |  | [Karl *et al.* 2011](#_ENREF_17) |
|  |  |  |  | ND4/CR | 0.00007-0.00151 |  | [Tillett *et al.* 2012b](#_ENREF_40) |
| Carcharhiniformes | Carcharhinidae | Blacktip Shark | *Carcharhinus limbatus* | CR | 0.00035-0.00282 | 0.00214 | [Keeney *et al.* 2005](#_ENREF_20) |
|  |  |  |  | CR | 0.00214 | 0.00226 | [Keeney & Heist 2006](#_ENREF_19) |
|  |  |  |  | ND4 | 0.00046-0.00712 |  | [Ovenden *et al.* 2011](#_ENREF_28) |
| Carcharhiniformes | Carcharhinidae | Sandbar Shark | *Carcharhinus plumbeus* | CR | 0.00161-0.00468 | 0.00475 | [Portnoy *et al.* 2010](#_ENREF_32) |
| Carcharhiniformes | Carcharhinidae | Dusky Shark | *Carcharhinus obscurus* | CR | 0.00089-0.00466 | 0.005 | [Benavides *et al.* 2011b](#_ENREF_3) |
| Carcharhiniformes | Carcharhinidae | Northern River Shark | *Glyphis garricki* | COXI | 0 |  | [Wynen *et al.* 2009](#_ENREF_46) |
|  |  |  |  | CR | 0 |  | [Wynen *et al.* 2009](#_ENREF_46) |
| Carcharhiniformes | Carcharhinidae | Speartooth Shark | *Glyphis glyphis* | COXI | 0 |  | [Wynen *et al.* 2009](#_ENREF_46) |
|  |  |  |  | CR | 0 |  | [Wynen *et al.* 2009](#_ENREF_46) |
| Carcharhiniformes | Carcharhinidae | Sharptooth Lemon Shark | *Negaprion acutidens* | CR | 0-0.00185 | 0.00056 | [Schultz *et al.* 2008](#_ENREF_37) |
| Carcharhiniformes | Carcharhinidae | Lemon Shark | *Negaprion brevirostris* | CR | 0-0.00099 | 0.00585 | [Schultz *et al.* 2008](#_ENREF_37) |
| Carcharhiniformes | Carcharhinidae | Milk Shark | *Rhizoprionodon acutus* | ND4 | 0.00209-0.00390 |  | [Ovenden *et al.* 2011](#_ENREF_28) |
| Carcharhiniformes | Carcharhinidae | Brazilian Sharpnose Shark | *Rhizoprionodon lalandii* | CR | 0.00484 |  | [Mendonça *et al.* 2009](#_ENREF_26) |
| Carcharhiniformes | Carcharhinidae | Caribbean Sharpnose Shark | *Rhizoprionodon porosus* | CR | 0.00278 |  | [Mendonça *et al.* 2011](#_ENREF_25) |
| Carcharhiniformes | Carcharhinidae | Whitetip Reef Shark | *Triaenodon obesus* | CR | 0-0.004 | 0.00213 | [Whitney *et al.* 2012](#_ENREF_45) |
| Carcharhiniformes | Sphyrnidae | Scalloped Hammerhead | *Sphyrna lewini* | CR | 0-0.0110 | 0.0130 | [Duncan *et al.* 2006](#_ENREF_10) |

Ahonen H, Harcourt R, Stow A (2009) Nuclear and mitochondrial DNA reveals isolation of imperilled grey nurse shark populations (*Carcharias taurus*). *Molecular Ecology* **18**, 4409-4421.

Benavides MT, Feldheim KA, Duffy CA*, et al.* (2011a) Phylogeography of the copper shark (*Carcharhinus brachyurus*) in the southern hemisphere: implications for the conservation of a coastal apex predator. *Marine and Freshwater Research* **62**, 861-869.

Benavides MT, Horn RL, Feldheim KA*, et al.* (2011b) Global phylogeography of the dusky shark *Carcharhinus obscurus*: implications for fisheries management and monitoring the shark fin trade. *Endangered Species Research* **14**, 13-22.

Blower DC, Pandolfi JM, Bruce BD, Gomez-Cabrera MdC, Ovenden JR (2012) Population genetics of Australian white sharks reveals fine-scale spatial structure, transoceanic dispersal events and low effective population sizes. *Marine Ecology Progress Series* **455**, 229-244.

Castillo-Páez A, Sosa-Nishizaki O, Sandoval-Castillo J, Galván-Magaña F, Rocha-Olivares A (2014) Strong Population Structure and Shallow Mitochondrial Phylogeny in the Banded Guitarfish, *Zapteryx exasperata* (Jordan y Gilbert, 1880), from the Northern Mexican Pacific. *Journal of Heredity* **105**, 91-100.

Castro A, Stewart B, Wilson S*, et al.* (2007) Population genetic structure of Earth's largest fish, the whale shark (*Rhincodon typus*). *Molecular Ecology* **16**, 5183-5192.

Chabot C, Allen L (2009) Global population structure of the tope (*Galeorhinus galeus*) inferred by mitochondrial control region sequence data. *Molecular Ecology* **18**, 545-552.

Chevolot M, Wolfs PH, Pálsson J, Rijnsdorp AD, Stam WT, Olsen JL (2007) Population structure and historical demography of the thorny skate (*Amblyraja radiata*, Rajidae) in the North Atlantic. *Marine Biology* **151**, 1275-1286.

Dudgeon C, Broderick D, Ovenden J (2009) IUCN classification zones concord with, but underestimate, the population genetic structure of the zebra shark *Stegostoma fasciatum* in the Indo‐West Pacific. *Molecular Ecology* **18**, 248-261.

Duncan K, Martin A, Bowen B, De Couet H (2006) Global phylogeography of the scalloped hammerhead shark (*Sphyrna lewini*). *Molecular Ecology* **15**, 2239-2251.

Frederico RG, Farias IP, Araújo MLGd, Charvet-Almeida P, Alves-Gomes JA (2012) Phylogeography and conservation genetics of the Amazonian freshwater stingray *Paratrygon aiereba* Müller & Henle, 1841 (Chondrichthyes: Potamotrygonidae). *Neotropical Ichthyology* **10**, 71-80.

Galván-Tirado C, Díaz-Jaimes P, García-de León FJ, Galván-Magaña F, Uribe-Alcocer M (2013) Historical demography and genetic differentiation inferred from the mitochondrial DNA of the silky shark (*Carcharhinus falciformis*) in the Pacific Ocean. *Fisheries Research* **147**, 36-46.

Geraghty PT, Williamson JE, Macbeth WG*, et al.* (2013) Population expansion and genetic structure in *Carcharhinus brevipinna* in the southern Indo-Pacific. *Plos One* **8**, e75169.

Griffiths AM, Sims DW, Cotterell SP*, et al.* (2010) Molecular markers reveal spatially segregated cryptic species in a critically endangered fish, the common skate (*Dipturus batis*). *Proceedings of the Royal Society B: Biological Sciences* **277**, 1497-1503.

Griffiths AM, Sims DW, Johnson A*, et al.* (2011) Levels of connectivity between longnose skate (*Dipturus oxyrinchus*) in the Mediterranean Sea and the north-eastern Atlantic Ocean. *Conservation Genetics* **12**, 577-582.

Hoelzel AR, Shivji MS, Magnussen J, Francis MP (2006) Low worldwide genetic diversity in the basking shark (*Cetorhinus maximus*). *Biology Letters* **2**, 639-642.

Karl S, Castro A, Lopez J, Charvet P, Burgess G (2011) Phylogeography and conservation of the bull shark (*Carcharhinus leucas*) inferred from mitochondrial and microsatellite DNA. *Conservation Genetics* **12**, 371-382.

Karl SA, Castro AL, Garla RC (2012) Population genetics of the nurse shark (*Ginglymostoma cirratum*) in the western Atlantic. *Marine Biology* **159**, 489-498.

Keeney D, Heist E (2006) Worldwide phylogeography of the blacktip shark (*Carcharhinus limbatus*) inferred from mitochondrial DNA reveals isolation of western Atlantic populations coupled with recent Pacific dispersal. *Molecular Ecology* **15**, 3669-3679.

Keeney D, Heupel M, Hueter R, Heist E (2005) Microsatellite and mitochondrial DNA analyses of the genetic structure of blacktip shark (*Carcharhinus limbatus*) nurseries in the northwestern Atlantic, Gulf of Mexico, and Caribbean Sea. *Molecular Ecology* **14**, 1911-1923.

Le Port A, Lavery S (2012) Population structure and phylogeography of the short-tailed stingray, *Dasyatis brevicaudata* (Hutton 1875), in the Southern Hemisphere. *Journal of Heredity* **103**, 174-185.

Lewallen EA, Anderson TW, Bohonak AJ (2007) Genetic structure of leopard shark (*Triakis semifasciata*) populations in California waters. *Marine Biology* **152**, 599-609.

Li N, Chen X, Sun D, Song N, Lin Q, Gao T (In press) Phylogeography and population structure of the red stingray, *Dasyatis akajei* inferred by mitochondrial control region. *Mitochondrial DNA* **0**, 1-9.

McLachlan (2011) *Comparative phylogeography of the catshark, Haploblepharus pictus and its nematode parasite, Proleptus*

*obtusus*, University of Stellenbosch.

Mendonça FF, Oliveira C, Gadig OB, Foresti F (2011) Phylogeography and genetic population structure of Caribbean sharpnose shark *Rhizoprionodon porosus*. *Reviews in Fish Biology and Fisheries* **21**, 799-814.

Mendonça FF, Oliveira C, Gadig OBF, Foresti F (2009) Populations analysis of the Brazilian Sharpnose Shark *Rhizoprionodon lalandii* (Chondrichthyes: Carcharhinidae) on the São Paulo coast, Southern Brazil: inferences from mt DNA sequences. *Neotropical Ichthyology* **7**, 213-216.

Murray BW, Wang JY, Yang S-C, Stevens JD, Fisk A, Svavarsson J (2008) Mitochondrial cytochrome b variation in sleeper sharks (Squaliformes: Somniosidae). *Marine Biology* **153**, 1015-1022.

Ovenden JR, Morgan JA, Street R*, et al.* (2011) Negligible evidence for regional genetic population structure for two shark species *Rhizoprionodon acutus* (Rüppell, 1837) and *Sphyrna lewini* (Griffith & Smith, 1834) with contrasting biology. *Marine Biology* **158**, 1497-1509.

Pasolini P, Ragazzini C, Zaccaro Z*, et al.* (2011) Quaternary geographical sibling speciation and population structuring in the Eastern Atlantic skates (suborder Rajoidea) *Raja clavata* and *R. straeleni*. *Marine Biology* **158**, 2173-2186.

Pereyra S, García G, Miller P, Oviedo S, Domingo A (2010) Low genetic diversity and population structure of the narrownose shark (*Mustelus schmitti*). *Fisheries Research* **106**, 468-473.

Phillips NM, Chaplin JA, Morgan DL, Peverell SC (2011) Population genetic structure and genetic diversity of three critically endangered *Pristis* sawfishes in Australian waters. *Marine Biology* **158**, 903-915.

Portnoy DS, McDowell JR, Heist EJ, Musick JA, Graves JE (2010) World phylogeography and male‐mediated gene flow in the sandbar shark, *Carcharhinus plumbeus*. *Molecular Ecology* **19**, 1994-2010.

Ramírez-Macías D, Vázquez-Juárez R, Galván-Magaña F, Munguía-Vega A (2007) Variations of the mitochondrial control region sequence in whale sharks (*Rhincodon typus*) from the Gulf of California, Mexico. *Fisheries Research* **84**, 87-95.

Sandoval-Castillo J, Rocha-Olivares A (2011) Deep mitochondrial divergence in Baja California populations of an aquilopelagic elasmobranch: the golden cownose ray. *Journal of Heredity* **102**, 269-274.

Sandoval-Castillo J, Rocha-Olivares A, Villavicencio-Garayzar C, Balart E (2004) Cryptic isolation of Gulf of California shovelnose guitarfish evidenced by mitochondrial DNA. *Marine Biology* **145**, 983-988.

Schluessel V, Broderick D, Collin S, Ovenden J (2010) Evidence for extensive population structure in the white‐spotted eagle ray within the Indo‐Pacific inferred from mitochondrial gene sequences. *Journal of zoology* **281**, 46-55.

Schultz J, Feldheim K, Gruber S, Ashley M, McGovern T, Bowen B (2008) Global phylogeography and seascape genetics of the lemon sharks (genus *Negaprion*). *Molecular Ecology* **17**, 5336-5348.

Stow A, Zenger K, Briscoe D*, et al.* (2006) Isolation and genetic diversity of endangered grey nurse shark (*Carcharias taurus*) populations. *Biology Letters* **2**, 308-311.

Tillett B, Meekan M, Broderick D, Field I, Cliff G, Ovenden J (2012a) Pleistocene isolation, secondary introgression and restricted contemporary gene flow in the pig-eye shark, *Carcharhinus amboinensis* across northern Australia. *Conservation Genetics* **13**, 99-115.

Tillett B, Meekan M, Field I, Thorburn D, Ovenden J (2012b) Evidence for reproductive philopatry in the bull shark *Carcharhinus leucas*. *Journal of Fish Biology* **80**, 2140-2158.

Trejo T (2005) *Global phylogeography of the thresher sharks (Alopias spp.) inferred from mitochondrial DNA control region sequences*, California State University Monterey Bay.

Veríssimo A, McDowell J, Graves J (2010) Global population structure of the spiny dogfish *Squalus acanthias*, a temperate shark with an antitropical distribution. *Molecular Ecology* **19**, 1651-1662.

Veríssimo A, McDowell J, Graves J (2012) Genetic population structure and connectivity in a commercially exploited and wide-ranging deepwater shark, the leafscale gulper (*Centrophorus squamosus*). *Marine and Freshwater Research* **63**, 505-512.

Veríssimo A, McDowell JR, Graves JE (2011) Population structure of a deep-water squaloid shark, the Portuguese dogfish (*Centroscymnus coelolepis*). *ICES Journal of Marine Science: Journal du Conseil* **68**, 555-563.

Whitney NM, Robbins WD, Schultz JK, Bowen BW, Holland KN (2012) Oceanic dispersal in a sedentary reef shark (*Triaenodon obesus*): genetic evidence for extensive connectivity without a pelagic larval stage. *Journal of Biogeography* **39**, 1144-1156.

Wynen L, Larson H, Thorburn D*, et al.* (2009) Mitochondrial DNA supports the identification of two endangered river sharks (*Glyphis glyphis* and *Glyphis garricki*) across northern Australia. *Marine and Freshwater Research* **60**, 554-562.
