# Supplementary material for: Mitogenomics of the Speartooth Shark challenges ten years of control region sequencing
Source: BMC Evol Biol. 2014 Nov 19;14:232. doi: 10.1186/s12862-014-0232-x (PMC4245800; doi:10.1186/s12862-014-0232-x)
Supplement: Additional file 5: Figure S1. — Maximum likelihood haplotype tree. [file 12862_2014_232_MOESM5_ESM.docx]

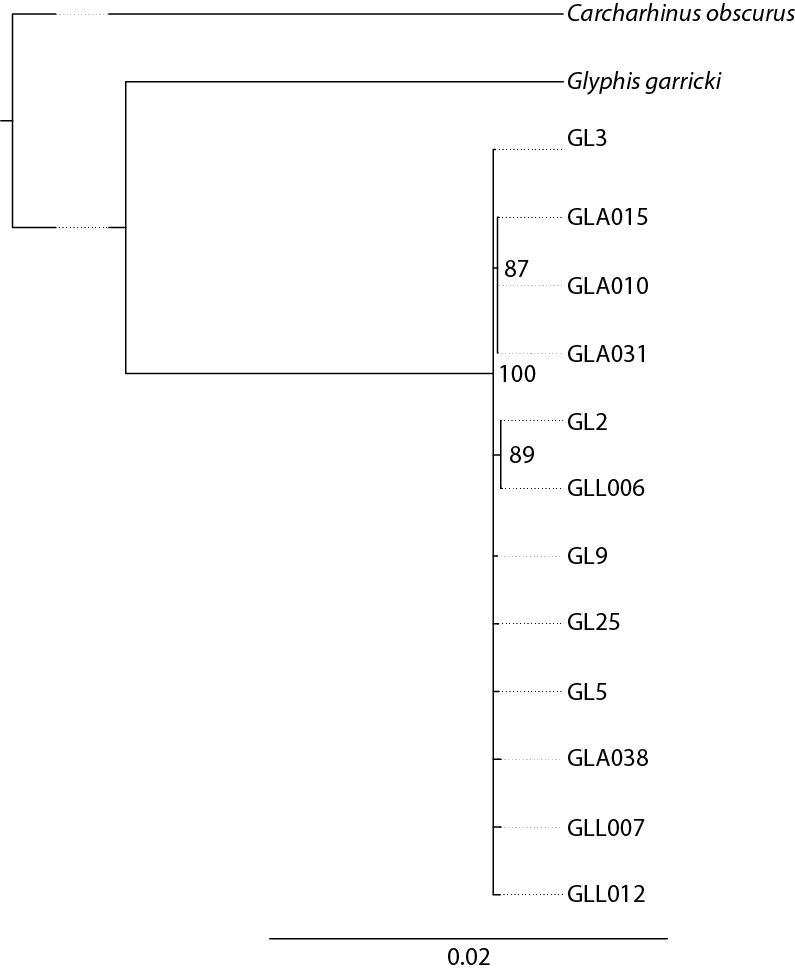


Figure S1. Maximum likelihood haplotype tree. Only bootstrap values above 0.75 are shown.

The haplotype tree was built using RAxML-HPC2 on XSEDE (v. 8.0.0) (Stamatakis, 2014) as implemented in the CIPRES Science Gateway (Miller, 2010) with default parameters and the Majority Rule Criterion to automatically stop bootstrapping. The congeneric species and closest match in Genbank *Glyphis garricki* (Accession KF646786.1) and second closest match in Genbank *Carcharhinus obscurus* (Accession KC470543.1) were used as outgroups.

Miller, M.A., Pfeiffer, W., and Schwartz, T. (2010) "Creating the CIPRES Science Gateway for inference of large phylogenetic trees" in Proceedings of the Gateway Computing Environments Workshop (GCE), 14 Nov. 2010, New Orleans, LA pp 1 - 8.

Stamatakis, A. (2014) RAxML Version 8: A tool for Phylogenetic Analysis and Post-Analysis of Large Phylogenies. Bioinformatics 10.1093/bioinformatics/btu033
